# Purely Cortical Anaplastic Ependymoma

**DOI:** 10.1155/2012/541431

**Published:** 2012-10-21

**Authors:** Flávio Ramalho Romero, Marco Antônio Zanini, Luis Gustavo Ducati, Roberto Bezerra Vital, Newton Moreira de Lima Neto, Roberto Colichio Gabarra

**Affiliations:** ^1^Division of Neurosurgery, Botucatu Medical School, São Paulo State University (UNESP), 18618-970 Botucatu, SP, Brazil; ^2^Departamento de Neurologia, Psiquiatria e Psicologia, (UNESP), Distrito de Rubião Júnior s/n, 18618-970 Botucatu, SP, Brazil; ^3^Hospital das Clínicas, São Paulo State University (UNESP), 18618-970 Botucatu, SP, Brazil

## Abstract

Ependymomas are glial tumors derived from ependymal cells lining the ventricles and the central canal of the spinal cord. It may occur outside the ventricular structures, representing the extraventicular form, or without any relationship of ventricular system, called ectopic ependymona. Less than fifteen cases of ectopic ependymomas were reported and less than five were anaplastic. We report a rare case of pure cortical ectopic anaplastic ependymoma.

## 1. Introduction

Ependymomas are tumors derived from ependymal cells lining the ventricles or from the central canal of the spinal cord [1, 2]. It represents 3%–9% of all neuroepithelial neoplasms, 6%–12% of all pediatric brain tumors, and almost one-third of all brain tumors in patients younger than 3 years [3]. Forty percent of ependymomas are supratentorial, while 60% are infratentorial in location [4]. Ependymomas may manifest at any age (documented age ranges from 1 month to 81 years) with no gender predilection. Besides posterior fossa ependymoma arises most often in children (mean age, 6 years), supratentorial ependymoma generally manifests in an older age group (mean age, 18–24 years) [3]. 

Although it is a lesion arising from the ventricular system, sometimes it has extension outside the ventricles, through the cerebral tissue, representing the extraventricular form. Also, they may occur outside the ventricular structures, without any relationship of ventricular system, representing the rare group of ectopic ependymona. Less than thirty cases of ectopic ependymomas were reported, and almost fifteen were purely cortical, and only five cases were anaplastic lesions (Table 1). 

We report a rare case of pure cortical ectopic anaplastic ependymoma. 

## 2. Case

**Figure 1:**
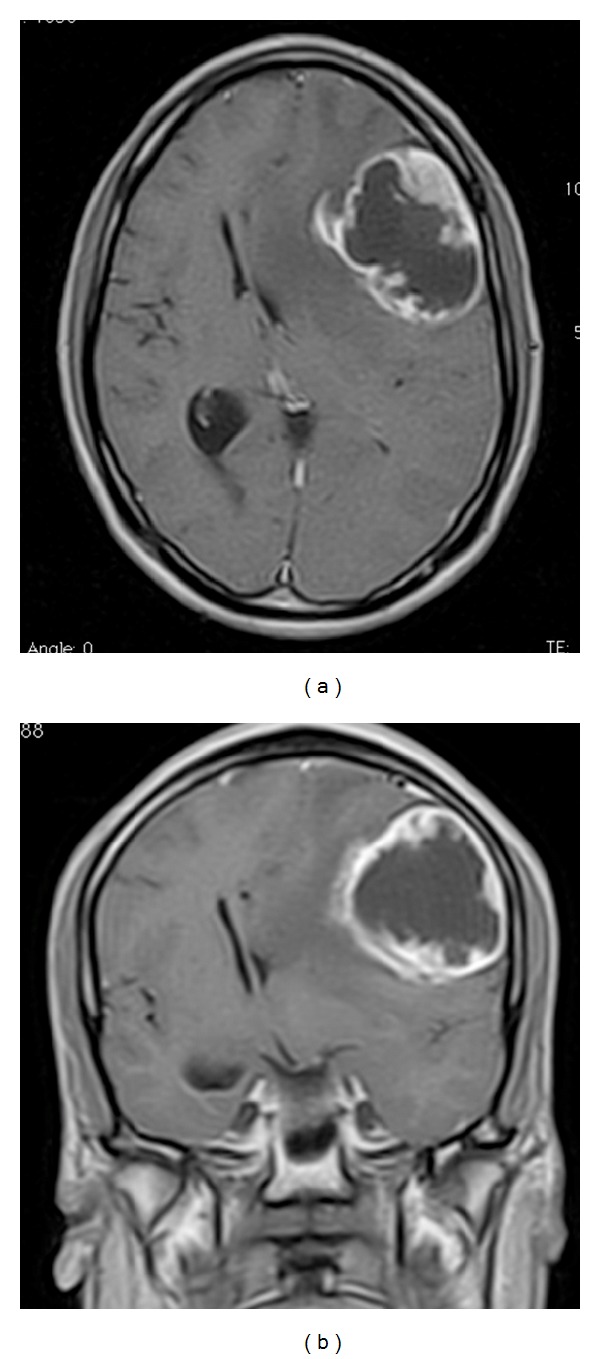
Preoperative MRI in axial (a) and coronal (b) view, showing extraventricular intraxial extensive lesion.

A 23-year-old male presented with seizures and progressive headache. Neurological examination showed right hemiparesis and motor aphasia. MRI (Figure 1) demonstrated a solid/cystic cortical expansive lesion in left frontal lobe with important edema and peripheral enhancing injection, without any relationship of lateral or third ventricle.

**Figure 2:**
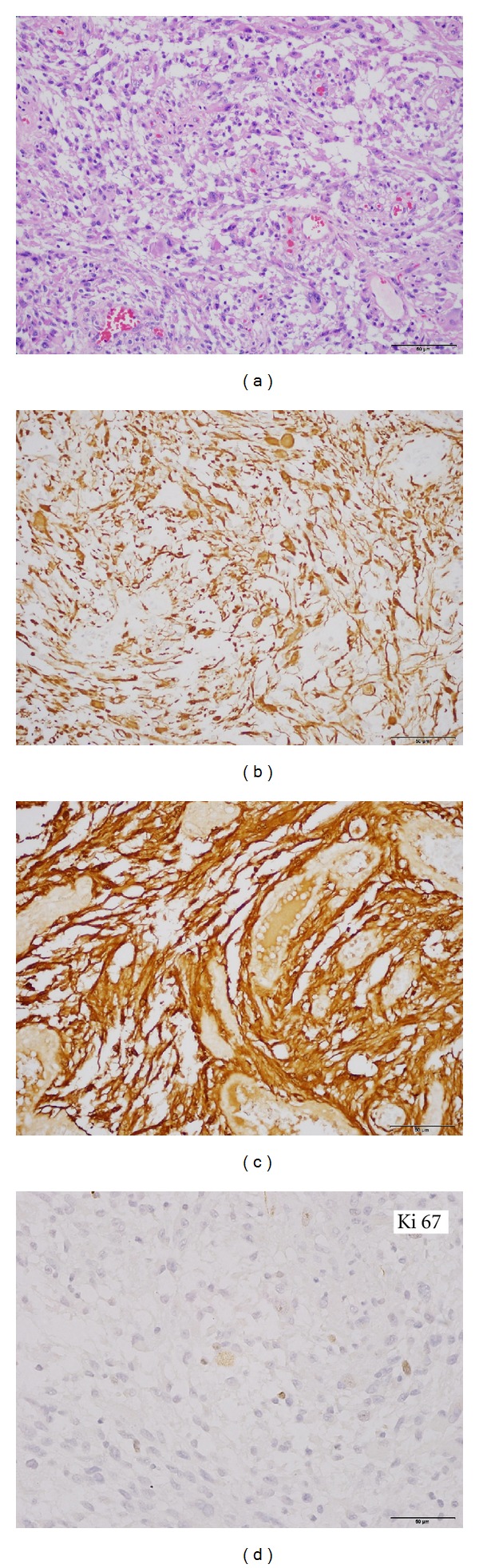
Histopathological and immunohistochemical (GFAP in(b), S-100 in (c)and KI-67 in (d)) features of the lesion. Hematoxylin and eosin stain (HE—(a)) showing perivascular pseudorosettes (anuclear zones formed by radially arranged tumor cell processes surrounding central blood vessels).

**Figure 3:**
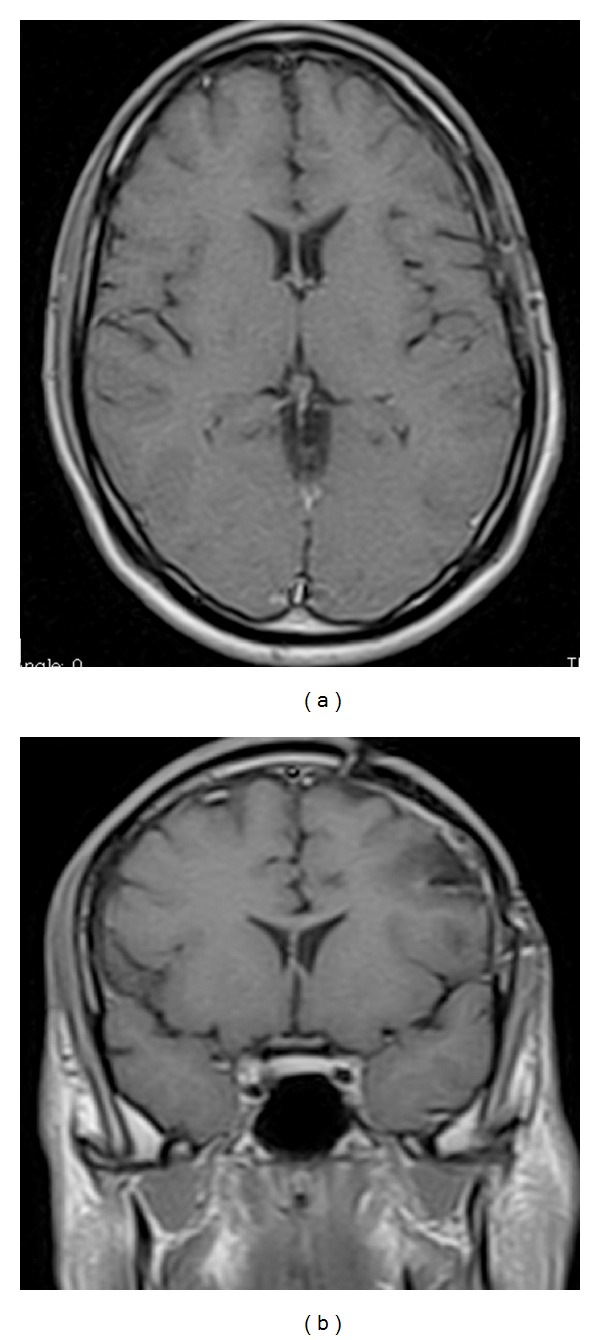
Postoperative MRI (after 4 years) in axial (a) and coronal (b) views, showing no residual tumor.

A left frontal craniotomy was performed allowing a microsurgical left frontal approach to the tumor. Total macroscopic removing was made and histological and imunohistoquimical examination confirmed typical findings of anaplastic ependymoma (Figure 2). After three months he recovered all neurological deficits, and new MRI showed no residual lesion. The patient was treated afterwards with external beam radiation. He has been stable with a followup of five years (Figure 3).

## 3. Discussion

Although approximately half of the supratentorial ependymomas arise from the wall of third or lateral ventricles and are purely intraventricular, the remaining has extension through adjacent cerebral tissue, representing extraventricular forms of ependymoma. Only few cases occur in distant places of the ventricular system, representing rare cases of ectopic lesions [10]. It is speculated that ectopic ependymomas may arise from embryonic rests of ependymal tissue trapped in the developing cerebral hemispheres [3]. 

Besides supratentorial ependymoma grow up of third or lateral ventricle, it is predominant involving the brain parenchyma at the diagnosis [6, 10]. Hamano et al. [7] reported that 83% of supratentorial ependymomas are in the cerebral parenchyma. Owing to its parenchymal location, the supratentorial ependymoma tends to be larger in size at the diagnosis. Roncaroli et al. [10] found that 94% of supratentorial tumors manifest with a size larger than 4 cm and often contain a cystic component [10, 12]. Despite their large size in the cerebral hemispheres, symptoms are relatively mild until a later stage of presentation [2, 3]. Symptoms of raised intracranial pressure such as headache and vomiting are common, whereas focal signs as limb weakness and seizures are less prevalent [3, 10]. 

The principal differential diagnosis of extraventricular supratentorial ependymoma should include astrocytoma (both low grade and glioblastoma multiforme), supratentorial primitive neuroectodermal tumor (PNET), ganglioglioma, gangliocytoma, and oligodendroglioma [4, 7, 10]. They have no typical images findings, but every lesion, with extension to the ventricular system is suspicious. They are iso- to slightly hypoattenuating to surrounding normal brain tissue at unenhanced CT [2, 6, 10, 12]. They are iso- to hypointense relative to normal white matter on unenhanced T1-weighted MR images and hyperintense on T2- and proton-density-weighted MR images. Foci of signal heterogeneity within a solid neoplasm represent methemoglobin, hemosiderin, necrosis, or calcification, that is very common in this tumor (40%–80% of cases) [3, 4, 6, 12]. Ependymomas can display variable contrast enhancement behavior but generally enhance moderately intensely at both CT and MR imaging, with central areas of necrosis [1, 2, 10]. 

Histologically, the tumor cells are characteristically organized in perivascular pseudorosettes and, less commonly, ependymal rosettes [3, 4]. Althought ependymomas are moderately cellular tumors with rare mitotic figures (World Health Organization (WHO) grade II lesions), our patient had a more aggressive tumor, classified as WHO grade III [3]. Less than five ectopic anaplastic ependymomas were reported previously. 

Prognostic factors of ependymomas that positively contribute to progression-free survival and longer survival are still elusive, even histologic characteristics [3, 7, 10, 12]. The 5-year progression-free rate for children overall is about 50% and 10-year survival rates for adults are 57.1% and 45%, respectively [3, 8, 16, 17]. Only total tumor resection is considered as a reliable prognostic factor for predicting longer survival time [2, 3, 12, 16]. Of patients with no radiologic evidence of residual tumor, 75% ± 15% will remain tumor free after 5 years as opposed to the group of patients with residual disease in which progression cannot be stopped [5, 9, 17]. 

Age at presentation is also a significant prognostic factor [5, 9, 17]. Patients younger than 3 years have a significantly worse outcome than older children or adults [8, 16, 17]. The last prognostic variable is the duration of symptoms before diagnosis. Patients with symptoms before diagnosis less than 1 month have a worse outcome than those with a more protracted course [16]. 

The best treatment is radical resection, because it appears that tumor resectibility is the most important factor associated with recurrence [2, 3, 17]. Pure cortical (ectopic) tumors are approached easier than lesions with ventricular extension, having better outcome. Postoperative radiation therapy must be administered in every case of partially resected ependymomas or anaplastic tumors. Chemotherapy and prophylactic craniospinal irradiation are not indicate as adjuvant treatment [5, 7, 8, 16].

Our patient was treated with radical surgery and postoperative radiation therapy, because their anaplastic grade tumor. There was no evidence of residual tumor at postoperative imaging. The patient had a good recovery of neurological symptoms, and after 5 years, he was tumor free at clinical and radiologic examination.

## Figures and Tables

**Table 1:** All reported cases of pure cortical supratentorial ependymomas.

Series	Cases	Presentation	Treatment	Followup (months)	Grade
Nakamizo et al., [5]	1	Seizure	S + RT	6	II
Davis et al., [2]	1	Seizures	S + RT	12	III
Alexiou et al., [6]	1	Headache, seizures	S + RT	?	III
Hamano et al., [7]	1	Headache	S + RT	18	III
Yadav et al., [8]	1	Hemiparesis, seizures	S	20	II
Ghani et al., [9]	1	Seizures, hemiparesis	S + RT	36	II
Roncaroli et al., [10]	3	Seizures	S	48	II
Saito et al., [11]	1	Seizures	S + RT	14	II
Ono et al., [12]	1	Seizures, headache	S + RT	18	II
Ehtesham et al., [3]	1	Seizures	S	12	II
Lehman et al., [13]	1	Seizures	S	5	II
Akyuz et al., [14]	1	Seizures, hemiparesis	S + RT	6	III
Goodkin et al., [15]	1	Seizures	S	?	II
Present case	1	Seizures, hemiparesis, and aphasia	S + RT	60	III

Legend: S: surgery, RT: radiotherapy.
